# (±)-3-(5-Amino-3-methyl-1-phenyl-1*H*-pyrazol-4-yl)-2-benzo­furan-1(3*H*)-one

**DOI:** 10.1107/S1600536813017479

**Published:** 2013-06-29

**Authors:** Rodolfo Moreno-Fuquen, Juan C. Castillo, Rodrigo Abonia, Javier Ellena, Juan C. Tenorio

**Affiliations:** a Departamento de Química, Facultad de Ciencias, Universidad del Valle, Apartado 25360, Santiago de Cali, Colombia; b Instituto de Física de São Carlos, IFSC, Universidade de São Paulo, USP, São Carlos, SP, Brazil

## Abstract

In the title compound, C_18_H_15_N_3_O_2_, the benzo­furan ring system is essentially planar, the rings making a dihedral angle of 0.57 (9)°. The phenyl, furan and benzene rings subtend dihedral angles of 47.07 (10), 85.76 (7) and 86.04 (7)°, respectively, with the pyrazole ring. In the crystal, mol­ecules are linked by weak N—H⋯N, N—H⋯O and C—H⋯O inter­actions, generating edge-fused *R*_4_^4^(20), and *R*_1_^2^(7) rings linked into sheets which are parallel to (010).

## Related literature

For biological and pharmacological properties of benzo­furan­ones, see: Yoganathan *et al.* (2003 ▶); Shode *et al.* (2002 ▶); Anderson *et al.* (2005 ▶); Puder *et al.* (2000 ▶); Nannei *et al.* (2006 ▶); Brady *et al.* (2000 ▶); Malpani *et al.* (2013 ▶). For the synthesis of diverse pyrazole derivatives, see: Abonia *et al.* (2010 ▶); Insuasty *et al.* (2012 ▶, 2013 ▶). For hydrogen bonding, see: Nardelli (1995 ▶) and for hydrogen-bond graph-set motifs, see: Etter (1990 ▶); Bernstein *et al.* (1995 ▶).
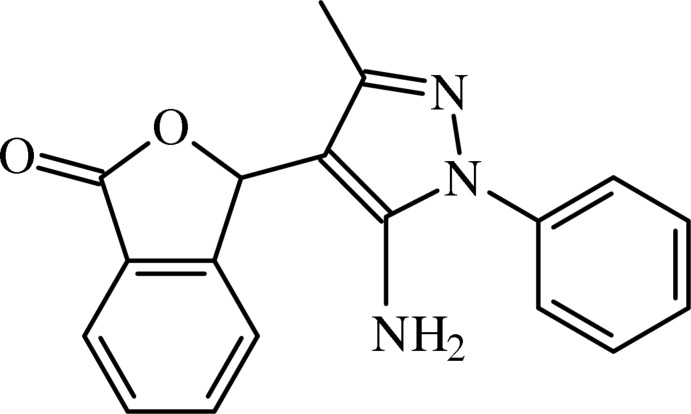


## Experimental

### 

#### Crystal data

C_18_H_15_N_3_O_2_
*M* *_r_* = 305.33
Monoclinic, 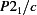

*a* = 10.0451 (2) Å
*b* = 15.0631 (5) Å
*c* = 12.2008 (4) Å
β = 123.257 (2)°
*V* = 1543.75 (8) Å^3^
*Z* = 4
Mo *K*α radiation
μ = 0.09 mm^−1^
*T* = 295 K
0.32 × 0.22 × 0.15 mm

#### Data collection

Nonius KappaCCD diffractometer
15125 measured reflections
3449 independent reflections
2264 reflections with *I* > 2σ(*I*)
*R*_int_ = 0.043

#### Refinement

*R*[*F* ^2^ > 2σ(*F* ^2^)] = 0.056
*wR*(*F* ^2^) = 0.173
*S* = 1.03
3449 reflections
212 parameters
H atoms treated by a mixture of independent and constrained refinement
Δρ_max_ = 0.29 e Å^−3^
Δρ_min_ = −0.31 e Å^−3^

### 

Data collection: *COLLECT* (Nonius, 2000 ▶); cell refinement: *SCALEPACK* (Otwinowski & Minor, 1997 ▶); data reduction: *DENZO* (Otwinowski & Minor, 1997 ▶) and *SCALEPACK*; program(s) used to solve structure: *SHELXS97* (Sheldrick, 2008 ▶); program(s) used to refine structure: *SHELXL97* (Sheldrick, 2008 ▶); molecular graphics: *ORTEP-3 for Windows* (Farrugia, 2012 ▶) and *Mercury* (Macrae *et al.*, 2006 ▶); software used to prepare material for publication: *WinGX* (Farrugia, 2012 ▶).

## Supplementary Material

Crystal structure: contains datablock(s) I, global. DOI: 10.1107/S1600536813017479/gg2119sup1.cif

Structure factors: contains datablock(s) I. DOI: 10.1107/S1600536813017479/gg2119Isup2.hkl

Supplementary material file. DOI: 10.1107/S1600536813017479/gg2119Isup3.cml

Additional supplementary materials:  crystallographic information; 3D view; checkCIF report

## Figures and Tables

**Table 1:** Hydrogen-bond geometry (Å, °)

*D*—H⋯*A*	*D*—H	H⋯*A*	*D*⋯*A*	*D*—H⋯*A*
N1—H1*A*⋯O1^i^	0.86	2.38	3.131 (2)	146
N1—H1*B*⋯N3^ii^	0.86	2.27	3.116 (2)	169
C8—H8⋯N3^ii^	1.013 (19)	2.51 (2)	3.484 (2)	159.9 (15)

Symmetry codes: (i) 
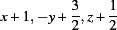
; (ii) 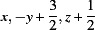.

**Fig. 1.**
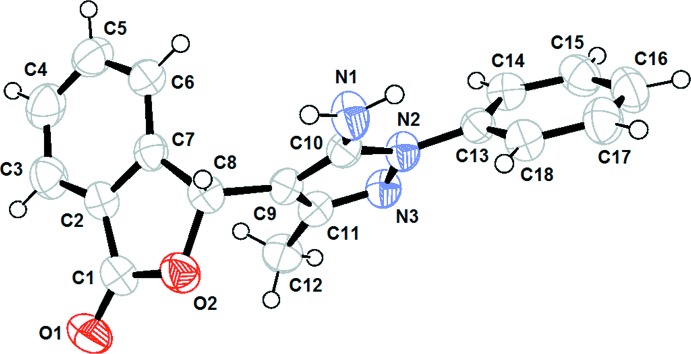
Molecular conformation and atom numbering scheme for the title compound (I) with displacement ellipsoids drawn at the 50% probability level. H atoms are shown as spheres of arbitrary radius.

**Fig. 2.**
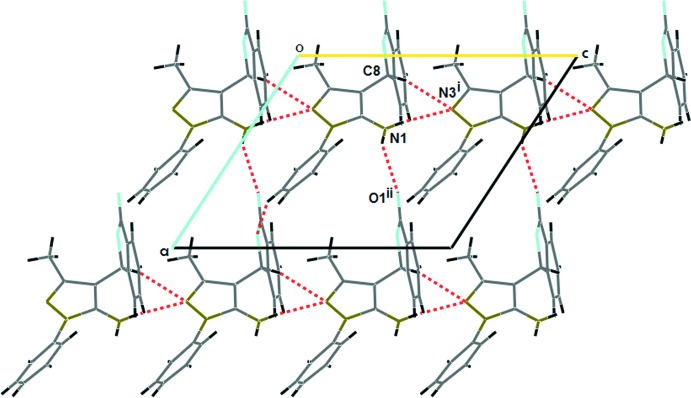
Part of the crystal structure of (I), showing the formation of chains of molecules which running parallel to (010). Symmetry code: (i) x + 1,-y + 1/2 + 1,+z + 1/2; (ii) x,-y + 1/2 + 1,+z + 1/2.

**Fig. 3.**
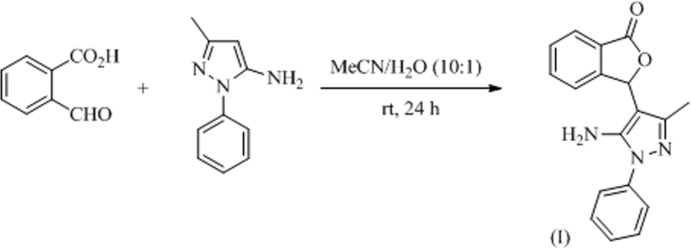
Reaction scheme.

## supplementary crystallographic information

### Comment

The title compound (±)-3-(5-amino-3-methyl-1-phenyl-1*H*-pyrazol-4-yl)isobenzofuran-1(3*H*)-one, (I), is part of the study of different crystal systems, associated with isobenzofuranones, which are an important class of synthetic and natural occurring products exhibiting diverse biological and pharmacological properties. Particularly, several of its 3-substitued derivatives are part of the framework of natural products such as fuscinarin with anti-HIV properties (Yoganathan *et al.*, 2003), typhaphthalide, a phenolic compound isolated from Typha capensis (Shode *et al.*, 2002), noscapine, with antitussive and anticancer properties (Anderson *et al.*, 2005), rubiginone-H, as antibiotic (Puder *et al.*, 2000), spirolaxine with antibacterial activity against Helicobacter pylori (Nannei *et al.*, 2006), cytosporone E with antibacterial properties (Brady *et al.*, 2000) and some synthetic spirolactones as inhibitors of the influenza virus type B (Malpani *et al.*, 2013). Continuing with our current studies on the use of pyrazoles for the synthesis of diverse pyrazole-derivatives with synthetic and biological interest (Abonia *et al.*, 2010; Insuasty *et al.*, 2012; Insuasty *et al.*, 2013), compound (I) was obtained from the reaction of 2-formylbenzoic acid with 5-amino-3-methyl-1-phenylpyrazole. In order to present the molecular conformation of (I) and its supramolecular behavior, the title compound was synthesized. The molecular structure of (I) is shown in Fig. 1. In the present molecule rings A (C2—C7) and B (O1—C1—C2—C7—C8) are planar showing a dihedral angle between them A/B = 0.57 (9)°. The phenyl, A and B rings form dihedral angles of 47.07 (10)°, 85.76 (7)° and 86.04 (7)° with the pyrazole ring respectively.

Further analysis showed that each molecule is linked to other molecules by weak N—H···N, N—H···O and C—H···O interactions (see table 1, Nardelli, 1995). These intermolecular contacts are explained in terms of the substructure shown in figure 2. The N3 atom in the molecule at (*x*,*y*,*z*) acts as hydrogen bond donor to pyrazolic N1 atom at (*x*,-*y* - 1/2,+*z* + 1/2). At the same time the N3 atom is linked to another molecule *via* N—H···O. Indeed, the N3 atom in the molecule at (*x*,*y*,*z*) acts as hydrogen bond donor to C=O O2 atom in the molecule at (*x* +1,-*y* - 1/2,+*z* + 1/2). Growth of the crystal is reinforced by the weak interaction C11—H11···N1, in which the C11 atom of the benzofuranone ring at (*x*,*y*,*z*) acts as hydrogen-bond donor to atom N1 in the molecule at (*x*,-*y* - 1/2,+*z* + 1/2). The combination of these intermolecular contacts generate edge-fused *R*^4^_4_(20), and *R*^2^_1_(7) (Fig. 2) ring motifs (Etter, 1990; Bernstein *et al.*, 1995), as sheets which stack parallel to (010).

### Experimental

Reagents and solvents for the synthesis were obtained from the Aldrich Chemical Co., and were used without additional purification. The 5-amino-3-methyl-1-phenyl-1*H*-pyrazole (117 mg, 0.68 mmol) and 2-formylbenzoic acid (103 mg, 0.69 mmol) were dissolved in a mixture of MeCN/H_2_O (10:1, 2 mL). The solution was stirred at room temperature for 24 h until the starting materials were not detected by TLC. Then, the solid formed was filtered and washed with cold MeCN (1 mL) without further purification (See scheme 2). White crystals of (I) suitable for single-crystal X-ray diffraction were grown by slow evaporation, at ambient temperature and in air, from a solution in ethanol (87% yield, m.p. 464 (1) K). MS (ESI+): m/*z* found: 306 [*M*+H]^+^, 328 [*M*+Na]^+^; elemental analysis found: C 71.13, H 5.01, N 13.69%; C_18_H_15_N_3_O_2_ requires: C 70.81, H 4.95, N 13.76%.

### Refinement

All H-atoms were positioned at geometrically idealized positions [N—H= 0.86 Å, C—H= 0.93 Å for aromatic, C—H= 0.96 Å for methyl group] and refined using a riding model approximation with *U*_iso_(H) constrained to 1.2 (N—H and aromatic) and to 1.5 (methyl) times *U*_eq_ of the respective parent atom. Coordinates for H11 were freely refined.

### Crystal data

**Table:**

C_18_H_15_N_3_O_2_	*F*(000) = 640
*M**_r_* = 305.33	*D*_x_ = 1.314 Mg m^−^^3^
Monoclinic, *P*2_1_/*c*	Melting point: 464(1) K
Hall symbol: -P 2ybc	Mo *K*α radiation, λ = 0.71073 Å
*a* = 10.0451 (2) Å	Cell parameters from 14996 reflections
*b* = 15.0631 (5) Å	θ = 2.6–27.5°
*c* = 12.2008 (4) Å	µ = 0.09 mm^−^^1^
β = 123.257 (2)°	*T* = 295 K
*V* = 1543.75 (8) Å^3^	Block, white
*Z* = 4	0.32 × 0.22 × 0.15 mm

### Data collection

**Table:**

Nonius KappaCCD diffractometer	2264 reflections with *I* > 2σ(*I*)
Radiation source: fine-focus sealed tube	*R*_int_ = 0.043
Graphite monochromator	θ_max_ = 27.5°, θ_min_ = 3.4°
CCD rotation images, thick slices scans	*h* = −13→12
15125 measured reflections	*k* = −19→19
3449 independent reflections	*l* = −15→12

### Refinement

**Table:**

Refinement on *F*^2^	Primary atom site location: structure-invariant direct methods
Least-squares matrix: full	Secondary atom site location: difference Fourier map
*R*[*F*^2^ > 2σ(*F*^2^)] = 0.056	Hydrogen site location: inferred from neighbouring sites
*wR*(*F*^2^) = 0.173	H atoms treated by a mixture of independent and constrained refinement
*S* = 1.03	*w* = 1/[σ^2^(*F*_o_^2^) + (0.1038*P*)^2^ + 0.1468*P*] where *P* = (*F*_o_^2^ + 2*F*_c_^2^)/3
3449 reflections	(Δ/σ)_max_ < 0.001
212 parameters	Δρ_max_ = 0.29 e Å^−^^3^
0 restraints	Δρ_min_ = −0.31 e Å^−^^3^

### Special details

**Table:**

Geometry. All esds (except the esd in the dihedral angle between two l.s. planes) are estimated using the full covariance matrix. The cell esds are taken into account individually in the estimation of esds in distances, angles and torsion angles; correlations between esds in cell parameters are only used when they are defined by crystal symmetry. An approximate (isotropic) treatment of cell esds is used for estimating esds involving l.s. planes.
Refinement. Refinement of *F*^2^ against ALL reflections. The weighted *R*-factor *wR* and goodness of fit *S* are based on *F*^2^, conventional *R*-factors *R* are based on *F*, with *F* set to zero for negative *F*^2^. The threshold expression of *F*^2^ > σ(*F*^2^) is used only for calculating *R*-factors(gt) *etc*. and is not relevant to the choice of reflections for refinement. *R*-factors based on *F*^2^ are statistically about twice as large as those based on *F*, and *R*- factors based on ALL data will be even larger.

### Fractional atomic coordinates and isotropic or equivalent isotropic displacement parameters (Å^2^)

**Table:**

	*x*	*y*	*z*	*U*_iso_*/*U*_eq_	
O2	−0.07121 (16)	0.81568 (8)	0.27256 (14)	0.0549 (4)	
N2	0.36978 (18)	0.72091 (10)	0.28921 (14)	0.0438 (4)	
N3	0.27961 (18)	0.77174 (10)	0.17675 (13)	0.0455 (4)	
C13	0.4909 (2)	0.66591 (12)	0.29898 (16)	0.0413 (4)	
O1	−0.28573 (17)	0.90333 (11)	0.17908 (17)	0.0733 (5)	
C11	0.1725 (2)	0.81116 (11)	0.19068 (17)	0.0433 (4)	
N1	0.38101 (19)	0.68232 (11)	0.48354 (14)	0.0532 (5)	
H1A	0.4582	0.6461	0.5063	0.064*	
H1B	0.3447	0.6890	0.5325	0.064*	
C7	0.1242 (2)	0.92069 (12)	0.39955 (16)	0.0426 (4)	
C10	0.3162 (2)	0.72929 (11)	0.36959 (16)	0.0402 (4)	
C14	0.5007 (2)	0.57697 (13)	0.33054 (18)	0.0500 (5)	
H14	0.4357	0.5534	0.3556	0.060*	
C9	0.1904 (2)	0.78783 (11)	0.31016 (16)	0.0411 (4)	
C2	−0.0212 (2)	0.96295 (12)	0.33066 (17)	0.0465 (5)	
C6	0.2622 (2)	0.96761 (14)	0.48389 (18)	0.0527 (5)	
H6	0.3608	0.9395	0.5306	0.063*	
C8	0.1023 (2)	0.82334 (12)	0.36679 (18)	0.0446 (4)	
C18	0.5921 (2)	0.70181 (13)	0.26633 (18)	0.0500 (5)	
H18	0.5900	0.7624	0.2506	0.060*	
C12	0.0453 (2)	0.86596 (14)	0.08062 (18)	0.0559 (5)	
H12A	0.0652	0.8701	0.0123	0.084*	
H12B	0.0454	0.9244	0.1122	0.084*	
H12C	−0.0564	0.8386	0.0467	0.084*	
C17	0.6953 (2)	0.64713 (15)	0.2575 (2)	0.0588 (5)	
H17	0.7606	0.6705	0.2326	0.071*	
C1	−0.1431 (2)	0.89583 (13)	0.2522 (2)	0.0528 (5)	
C16	0.7027 (3)	0.55779 (16)	0.2851 (2)	0.0637 (6)	
H16	0.7713	0.5210	0.2772	0.076*	
C15	0.6087 (3)	0.52322 (14)	0.3245 (2)	0.0603 (5)	
H15	0.6175	0.4636	0.3471	0.072*	
C3	−0.0347 (3)	1.05330 (14)	0.3422 (2)	0.0609 (6)	
H3	−0.1330	1.0816	0.2950	0.073*	
C5	0.2482 (3)	1.05762 (15)	0.4961 (2)	0.0646 (6)	
H5	0.3388	1.0907	0.5529	0.077*	
C4	0.1022 (3)	1.09962 (15)	0.4257 (2)	0.0676 (6)	
H4	0.0966	1.1605	0.4351	0.081*	
H8	0.130 (2)	0.7852 (12)	0.4451 (19)	0.049 (5)*	

### Atomic displacement parameters (Å^2^)

**Table:**

	*U*^11^	*U*^22^	*U*^33^	*U*^12^	*U*^13^	*U*^23^
O2	0.0481 (8)	0.0467 (8)	0.0721 (9)	−0.0027 (6)	0.0345 (7)	−0.0020 (6)
N2	0.0513 (9)	0.0464 (8)	0.0380 (7)	0.0083 (7)	0.0272 (7)	0.0052 (6)
N3	0.0530 (10)	0.0484 (9)	0.0372 (8)	0.0065 (7)	0.0262 (7)	0.0059 (6)
C13	0.0418 (10)	0.0467 (10)	0.0363 (8)	0.0021 (8)	0.0219 (8)	−0.0011 (7)
O1	0.0421 (9)	0.0777 (11)	0.0866 (11)	0.0030 (7)	0.0267 (8)	−0.0077 (8)
C11	0.0482 (11)	0.0401 (10)	0.0405 (9)	−0.0007 (8)	0.0237 (8)	0.0005 (7)
N1	0.0624 (11)	0.0635 (10)	0.0424 (9)	0.0210 (8)	0.0344 (8)	0.0143 (7)
C7	0.0471 (11)	0.0470 (10)	0.0400 (9)	0.0025 (8)	0.0280 (8)	0.0023 (7)
C10	0.0456 (10)	0.0407 (10)	0.0363 (9)	−0.0006 (8)	0.0237 (8)	0.0002 (7)
C14	0.0509 (12)	0.0484 (11)	0.0542 (11)	0.0019 (9)	0.0310 (10)	0.0035 (8)
C9	0.0461 (11)	0.0398 (9)	0.0401 (9)	0.0003 (8)	0.0253 (8)	−0.0008 (7)
C2	0.0458 (11)	0.0464 (11)	0.0514 (10)	0.0035 (8)	0.0292 (9)	0.0017 (8)
C6	0.0486 (12)	0.0621 (13)	0.0449 (10)	0.0009 (10)	0.0241 (9)	−0.0054 (9)
C8	0.0450 (11)	0.0462 (10)	0.0458 (10)	0.0033 (8)	0.0270 (9)	0.0059 (8)
C18	0.0513 (12)	0.0520 (11)	0.0476 (10)	−0.0024 (9)	0.0278 (9)	0.0015 (8)
C12	0.0569 (13)	0.0578 (12)	0.0462 (10)	0.0067 (10)	0.0239 (10)	0.0087 (9)
C17	0.0508 (12)	0.0746 (15)	0.0594 (12)	0.0038 (11)	0.0355 (10)	0.0053 (10)
C1	0.0454 (12)	0.0571 (12)	0.0591 (11)	0.0042 (10)	0.0306 (10)	0.0026 (9)
C16	0.0555 (13)	0.0760 (16)	0.0649 (13)	0.0159 (11)	0.0365 (11)	0.0022 (11)
C15	0.0642 (14)	0.0516 (12)	0.0645 (13)	0.0115 (10)	0.0349 (11)	0.0059 (10)
C3	0.0590 (14)	0.0527 (13)	0.0714 (13)	0.0126 (10)	0.0360 (12)	0.0047 (10)
C5	0.0663 (14)	0.0635 (14)	0.0643 (13)	−0.0121 (12)	0.0360 (12)	−0.0164 (11)
C4	0.0804 (17)	0.0472 (12)	0.0795 (15)	0.0007 (12)	0.0465 (14)	−0.0080 (11)

### Geometric parameters (Å, º)

**Table:**

O2—C1	1.358 (2)	C2—C3	1.383 (3)
O2—C8	1.475 (2)	C2—C1	1.467 (3)
N2—C10	1.359 (2)	C6—C5	1.380 (3)
N2—N3	1.388 (2)	C6—H6	0.9300
N2—C13	1.422 (2)	C8—H8	1.013 (19)
N3—C11	1.319 (2)	C18—C17	1.375 (3)
C13—C14	1.382 (3)	C18—H18	0.9300
C13—C18	1.389 (2)	C12—H12A	0.9600
O1—C1	1.208 (2)	C12—H12B	0.9600
C11—C9	1.412 (2)	C12—H12C	0.9600
C11—C12	1.495 (3)	C17—C16	1.379 (3)
N1—C10	1.365 (2)	C17—H17	0.9300
N1—H1A	0.8600	C16—C15	1.375 (3)
N1—H1B	0.8600	C16—H16	0.9300
C7—C2	1.377 (3)	C15—H15	0.9300
C7—C6	1.384 (3)	C3—C4	1.371 (3)
C7—C8	1.504 (3)	C3—H3	0.9300
C10—C9	1.376 (2)	C5—C4	1.381 (3)
C14—C15	1.388 (3)	C5—H5	0.9300
C14—H14	0.9300	C4—H4	0.9300
C9—C8	1.489 (2)		
			
C1—O2—C8	110.83 (14)	C9—C8—C7	115.86 (15)
C10—N2—N3	111.11 (13)	O2—C8—H8	106.9 (11)
C10—N2—C13	130.77 (14)	C9—C8—H8	107.7 (10)
N3—N2—C13	117.96 (13)	C7—C8—H8	112.2 (11)
C11—N3—N2	104.90 (13)	C17—C18—C13	119.47 (18)
C14—C13—C18	120.37 (17)	C17—C18—H18	120.3
C14—C13—N2	121.01 (16)	C13—C18—H18	120.3
C18—C13—N2	118.44 (16)	C11—C12—H12A	109.5
N3—C11—C9	111.71 (16)	C11—C12—H12B	109.5
N3—C11—C12	119.26 (15)	H12A—C12—H12B	109.5
C9—C11—C12	128.82 (17)	C11—C12—H12C	109.5
C10—N1—H1A	120.0	H12A—C12—H12C	109.5
C10—N1—H1B	120.0	H12B—C12—H12C	109.5
H1A—N1—H1B	120.0	C18—C17—C16	120.47 (18)
C2—C7—C6	120.81 (18)	C18—C17—H17	119.8
C2—C7—C8	109.70 (16)	C16—C17—H17	119.8
C6—C7—C8	129.49 (17)	O1—C1—O2	120.95 (19)
N2—C10—N1	122.01 (15)	O1—C1—C2	130.16 (19)
N2—C10—C9	106.96 (14)	O2—C1—C2	108.88 (16)
N1—C10—C9	130.99 (15)	C15—C16—C17	119.97 (19)
C13—C14—C15	119.29 (18)	C15—C16—H16	120.0
C13—C14—H14	120.4	C17—C16—H16	120.0
C15—C14—H14	120.4	C16—C15—C14	120.29 (19)
C10—C9—C11	105.30 (15)	C16—C15—H15	119.9
C10—C9—C8	126.36 (15)	C14—C15—H15	119.9
C11—C9—C8	128.18 (16)	C4—C3—C2	117.51 (19)
C7—C2—C3	121.57 (18)	C4—C3—H3	121.2
C7—C2—C1	107.81 (16)	C2—C3—H3	121.2
C3—C2—C1	130.63 (18)	C6—C5—C4	121.3 (2)
C5—C6—C7	117.50 (19)	C6—C5—H5	119.3
C5—C6—H6	121.2	C4—C5—H5	119.3
C7—C6—H6	121.2	C3—C4—C5	121.3 (2)
O2—C8—C9	111.06 (14)	C3—C4—H4	119.4
O2—C8—C7	102.78 (14)	C5—C4—H4	119.4
			
C10—N2—N3—C11	0.49 (19)	C1—O2—C8—C9	−124.84 (16)
C13—N2—N3—C11	176.40 (15)	C1—O2—C8—C7	−0.32 (18)
C10—N2—C13—C14	45.9 (3)	C10—C9—C8—O2	−131.54 (18)
N3—N2—C13—C14	−129.02 (17)	C11—C9—C8—O2	53.7 (2)
C10—N2—C13—C18	−138.91 (19)	C10—C9—C8—C7	111.7 (2)
N3—N2—C13—C18	46.1 (2)	C11—C9—C8—C7	−63.1 (2)
N2—N3—C11—C9	0.29 (19)	C2—C7—C8—O2	−0.28 (17)
N2—N3—C11—C12	−174.89 (15)	C6—C7—C8—O2	179.67 (16)
N3—N2—C10—N1	176.82 (16)	C2—C7—C8—C9	121.01 (17)
C13—N2—C10—N1	1.6 (3)	C6—C7—C8—C9	−59.0 (2)
N3—N2—C10—C9	−1.07 (19)	C14—C13—C18—C17	4.1 (3)
C13—N2—C10—C9	−176.30 (17)	N2—C13—C18—C17	−171.06 (16)
C18—C13—C14—C15	−2.4 (3)	C13—C18—C17—C16	−2.3 (3)
N2—C13—C14—C15	172.66 (17)	C8—O2—C1—O1	−179.17 (17)
N2—C10—C9—C11	1.17 (19)	C8—O2—C1—C2	0.8 (2)
N1—C10—C9—C11	−176.46 (18)	C7—C2—C1—O1	179.0 (2)
N2—C10—C9—C8	−174.57 (16)	C3—C2—C1—O1	−0.7 (4)
N1—C10—C9—C8	7.8 (3)	C7—C2—C1—O2	−0.9 (2)
N3—C11—C9—C10	−0.9 (2)	C3—C2—C1—O2	179.40 (18)
C12—C11—C9—C10	173.66 (17)	C18—C17—C16—C15	−1.3 (3)
N3—C11—C9—C8	174.71 (17)	C17—C16—C15—C14	3.1 (3)
C12—C11—C9—C8	−10.7 (3)	C13—C14—C15—C16	−1.2 (3)
C6—C7—C2—C3	0.5 (3)	C7—C2—C3—C4	−0.4 (3)
C8—C7—C2—C3	−179.57 (16)	C1—C2—C3—C4	179.19 (19)
C6—C7—C2—C1	−179.22 (16)	C7—C6—C5—C4	−0.8 (3)
C8—C7—C2—C1	0.73 (19)	C2—C3—C4—C5	−0.2 (3)
C2—C7—C6—C5	0.1 (3)	C6—C5—C4—C3	0.8 (3)
C8—C7—C6—C5	−179.82 (17)		

### Hydrogen-bond geometry (Å, º)

**Table:**

*D*—H···*A*	*D*—H	H···*A*	*D*···*A*	*D*—H···*A*
N1—H1*A*···O1^i^	0.86	2.38	3.131 (2)	146
N1—H1*B*···N3^ii^	0.86	2.27	3.116 (2)	169
C8—H8···N3^ii^	1.013 (19)	2.51 (2)	3.484 (2)	159.9 (15)

Symmetry codes: (i) *x*+1, −*y*+3/2, *z*+1/2; (ii) *x*, −*y*+3/2, *z*+1/2.

## References

[bb1] Abonia, R., Castillo, J., Insuasty, B., Quiroga, J., Nogueras, M. & Cobo, J. (2010). *Eur. J. Org. Chem.* **33**, 6454–6463.

[bb2] Anderson, J. T., Ting, A. E., Boozer, S., Brunden, K. R., Crumrine, C., Danzig, J., Dent, T., Faga, L., Harrington, J. J., Hodnick, W. F., Murphy, S. M., Pawlowski, G., Perry, R., Raber, A., Rundlett, S. E., Stricker-Krongrad, A., Wang, J. & Bennani, Y. L. (2005). *J. Med. Chem.* **48**, 7096–7098. 10.1021/jm050674q 16279766

[bb3] Bernstein, J., Davis, R. E., Shimoni, L. & Chang, N.-L. (1995). *Angew. Chem. Int. Ed. Engl.* **34**, 1555–1573.

[bb4] Brady, S. F., Wagenaar, M. M., Singh, M. P., Janson, J. E. & Clardy, J. (2000). *Org. Lett.* **2**, 4043–4046. 10.1021/ol006680s 11112639

[bb5] Etter, M. (1990). *Acc. Chem. Res.* **23**, 120–126.

[bb6] Farrugia, L. J. (2012). *J. Appl. Cryst.* **45**, 849–854.

[bb7] Insuasty, B., Chamizo, L., Muñoz, J., Tigreros, A., Quiroga, J., Abonia, R., Nogueras, M. & Cobo, J. (2012). *Arch. Pharm.* **345**, 275–286. 10.1002/ardp.201100170 22105771

[bb8] Insuasty, H., Insuasty, B., Castro, E., Quiroga, J. & Abonia, R. (2013). *Tetrahedron Lett.* **54**, 1722–1725.

[bb9] Macrae, C. F., Edgington, P. R., McCabe, P., Pidcock, E., Shields, G. P., Taylor, R., Towler, M. & van de Streek, J. (2006). *J. Appl. Cryst.* **39**, 453–457.

[bb10] Malpani, Y., Achary, R., Kim, S. Y., Jeong, H. C., Kim, P., Han, S. B., Kim, M., Lee, C.-K., Kim, J. N. & Jung, Y.-S. (2013). *Eur. J. Med. Chem.* **62**, 534–544. 10.1016/j.ejmech.2013.01.015 23419738

[bb11] Nannei, R., Dallavalle, S., Merlini, L., Bava, A. & Nasini, G. (2006). *J. Org. Chem.* **71**, 6277–6280. 10.1021/jo060839i 16872220

[bb12] Nardelli, M. (1995). *J. Appl. Cryst.* **28**, 659.

[bb13] Nonius (2000). *COLLECT* Nonius BV, Delft, The Netherlands.

[bb14] Otwinowski, Z. & Minor, W. (1997). *Methods in Enzymology*, Vol. 276, *Macromolecular Crystallography*, Part A, edited by C. W. Carter Jr & R. M. Sweet, pp. 307-326. New York: Academic Press.

[bb15] Puder, C., Zeeck, A. & Beil, W. J. (2000). *J. Antibiot.* **53**, 329–336. 10.7164/antibiotics.53.329 10866213

[bb16] Sheldrick, G. M. (2008). *Acta Cryst.* A**64**, 112–122. 10.1107/S0108767307043930 18156677

[bb17] Shode, F. O., Mahomed, A. S. & Rogers, C. B. (2002). *Phytochemistry*, **61**, 955–957. 10.1016/s0031-9422(02)00439-9 12453524

[bb18] Yoganathan, K., Rossant, C., Ng, S., Huang, Y., Butler, M. S. & Buss, A. D. (2003). *J. Nat. Prod.* **66**, 1116–1117. 10.1021/np030146m 12932138

